# Deep Proteomic Profiles of the Antarctic Diatom *Fragilariopsis Cylindrus* Under Varying Iron and Manganese Conditions

**DOI:** 10.1002/pmic.70109

**Published:** 2026-02-13

**Authors:** Loay J. Jabre, Elden Rowland, J. Scott P. McCain, Erin M. Bertrand

**Affiliations:** ^1^ Department of Biology Dalhousie University Halifax Nova Scotia Canada; ^2^ Department of Biology Mount Allison Univesity Sackville New Brunswick Canada; ^3^ Department of Biology Massachusetts Institute of Technology Cambridge Massachusetts USA; ^4^ Department of Earth, Atmospheric, and Planetary Science Massachusetts Institute of Technology Cambridge Massachusetts USA

**Keywords:** data independent acquisition, phytoplankton, Southern Ocean, trace metals

## Abstract

*Fragilariopsis cylindrus* is a key diatom in the Southern Ocean, where low iron and manganese availability constrain primary production and biogeochemical activity. The molecular mechanisms used by polar diatoms, including *F. cylindrus*, to cope with trace metal limitations remain largely unexplored. Here we present phenotypic characterizations and proteomic profiles of *F. cylindrus* grown under controlled iron (low, medium, high) and manganese (low, high) conditions that reflect those observed in the Southern Ocean. Using data‐independent acquisition mass spectrometry, we measured over 8000 unique proteins capturing diverse metabolic responses, including those related to photosynthesis, elemental transport, and intracellular trafficking. We confirm consistent expression of canonical iron stress proteins (e.g., phytotransferrin) under low iron, and identify additional candidate biomarkers for iron and manganese stress that could be explored in future laboratory and field experiments. Our data also support the notion that one flavodoxin isoform in *F. cylindrus* is iron responsive and one is not, and show that PsaE, a protein associated with the iron‐rich photosystem‐I, is upregulated under low iron. Altogether, this dataset is among the most comprehensive proteomic characterizations of trace metal physiology in polar diatoms to date, providing a foundation for connecting molecular responses to trace metal availability and ocean biogeochemistry.

**Abbreviations**
F. cylindrus: Fragilariopsis cylindrus
Fe: iron
F_v_/F_m_
: variable fluorescence over maximum fluorescence
HCl: hydrochloric acid
Mn: manganese
UPLC: ultra performance liquid chromatography.

## 1 Main Text

Phytoplankton are a diverse group of photosynthetic microorganisms inhabiting the sunlit layers of the ocean. They perform half of all global photosynthesis [1], and in doing so, they form the foundation of marine food webs, influence elemental cycling [2], and contribute to the long‐term sequestration of atmospheric carbon in the deep ocean [3]. The Southern Ocean surrounding the Antarctic continent harbors intense seasonal phytoplankton blooms that are integral to Earth system processes. Phytoplankton growth in much of this region is, however, limited by low iron availability [5, 6], and recent evidence suggests limitation or co‐limitation by low manganese availability in some Southern Ocean locations [7, 8, 9]. While both iron and manganese are essential micronutrients involved in various cellular processes such as respiration, photosynthesis, nitrogen metabolism, and superoxide mediation, cellular requirements for iron are generally higher than for manganese [10].

**FIGURE 1:**
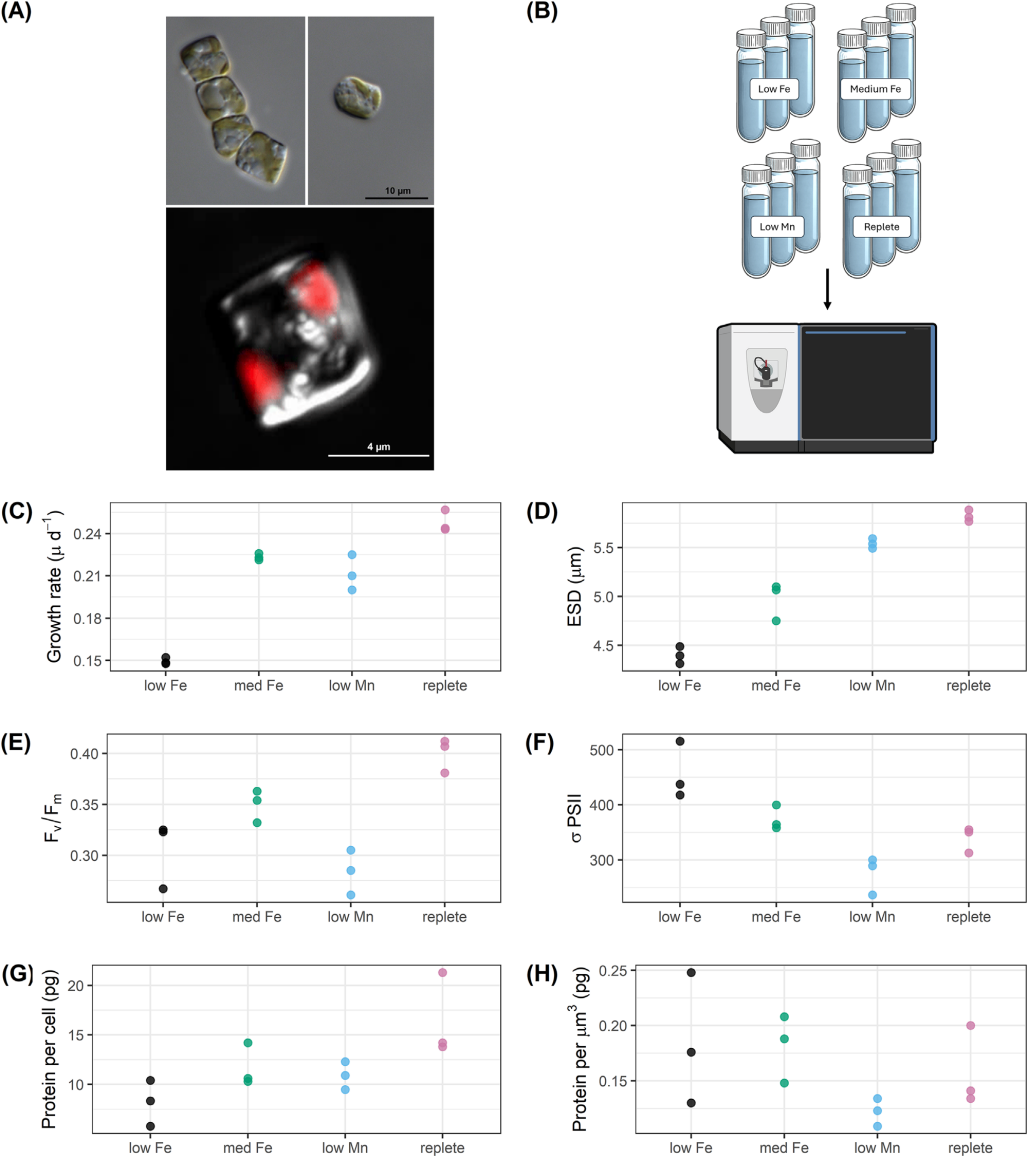
Experimental design and culture metadata. (**A)** top: light micrographs of *Fragilariopsis cylindrus* cells in a chain and solitary formation, bottom: merged DIC + fluorescence micrograph showing chlorophyll‐a fluorescence (red) marking chloroplast locations within the cell. (**B)** Schematic of 28 mL polycarbonate vials with treatment replicates. (**C)** Growth rates calculated as in [22]. (**D)** Estimated Spherical Diameter (ESD) measured via flow cytometry. (**E)** Photochemical efficiency of PSII (F_v_/F_m_), unitless. (**F)** Relative functional absorption cross‐section of PSII (*σ*PSII), unitless. (**G)** Total protein (picogram; pg) per cell. (**H)** Total protein (picogram; pg) per µm^3^ of cell volume. In C–H, each point represents one biological replicate. The low Fe, med Fe and replete data in C–F are replotted from [22].

Diatoms are a globally distributed group of phytoplankton that require silica to build their frustules (silica‐based cell wall structures), linking them to global silica cycles and other associated elementals like zinc [11, 12, 13, 14]. Diatoms alone contribute up to 40% of ocean primary productivity, and play a key role in the ocean's biogeochemical functions, including transporting carbon from the surface to the deep ocean, where it can be stored for hundreds to thousands of years [1, 15, 16]. In the Southern Ocean, diatoms are among the most abundant groups of phytoplankton, with *Fragilariopsis cylindrus* (Figure 1A) being a numerically dominant species [17]. This diatom has become a model system for cold‐adapted phytoplankton, and its fully sequenced genome [18] provides a useful resource for investigating the molecular mechanisms underpinning phytoplankton growth in polar regions.

Previous physiological and growth experiments on *F. cylindrus* have yielded important insights into its responses to trace metal limitation and other environmental variables [19, 20, 21, 22]. However, molecular characterizations of this diatom, and polar phytoplankton species in general, remain scarce. Lyon et al. (2011) [23] measured 110 *F. cylindrus* proteins to investigate dimethyl sulfoniopropionate (DMSP) biosynthesis, and Kennedy et al. (2019) [24] conducted a more comprehensive proteomics experiment investigating phytoplankton physiology during extended periods of darkness during Antarctic winters. To our knowledge, these are the only published *F. cylindrus* proteomics datasets, and a proteomic characterization under environmentally relevant iron and manganese conditions remains lacking. Filling this gap is crucial for advancing our understanding of polar phytoplankton adaptations, and for identifying protein biomarkers of iron and manganese limitation that inform phytoplankton physiological status and roles in biogeochemical processes.

Here, we present deep proteomic profiles of *F. cylindrus* cultivated under conditions that mimic key micronutrient regimes in the Southern Ocean: from severe iron limitation to iron‐replete, as well as manganese‐limiting and replete (Figure 1B). Our dataset comprises 8252 unique proteins across four distinct treatments, with three highly reproducible biological replicates in each treatment. Importantly, we also provide associated phenotypic data, like cell size, photophysiology, and growth rates (Figure 1C–H), offering a system‐level view of how *F. cylindrus* responds to varied micronutrient conditions. Together, these data can be used to inform cellular‐scale models of resource allocation [25], improve physiological parameterization in ecosystem models, and aid in the interpretation of metaproteomic measurements from the field.

To generate the dataset, we used *F. cylindrus* cultures from Jabre and Bertrand (2020) [22] in addition to cultures grown as part of the same experiment but not described in [22]. Briefly, biological triplicates of *F. cylindrus* (NCMA 1102) were grown under trace‐metal‐clean conditions in EDTA buffered Aquil* media containing the following iron and manganese treatments: (1) low iron (low Fe: 15 nM Fe + 48 nM Mn); (2) medium iron (med Fe: 35 nM Fe + 48 nM Mn); (3) low manganese (low Mn: 500 nM Fe + no added Mn); (4) replete (replete: 500 nM Fe + 48 nM Mn). For all treatments, temperature was kept at 3°C, and constant light was supplied at 50 µmol of photosynthetically active radiation m^−2^ s^−1^. Cells were harvested on 0.2 µm polycarbonate filters using gentle vacuum, then stored at −80°C until further processing.

Cultures grown under low and medium iron, and low manganese, had significantly lower growth rates and photosystem II efficiency (F_v_/F_m_) compared to the replete treatment (Figures 1C, E). This is indicative of iron and manganese stress, reflecting seasonally low iron and manganese conditions in the Southern Ocean.

Sample processing began with protein extraction, where 650 µL of lysis buffer (2% SDS, 0.1 M Tris/HCl pH 7.5, 5 mM EDTA) was added to each sample, followed by a 10‐min incubation on ice, and a further 15‐min incubation at 95°C and 350 RPM. Samples were then sonicated on ice for 1 min (50 % amplitude, 125 W) using a Q125 microprobe (QSonica), and incubated at room temperature for 30 min. Filters were removed and the protein‐containing lysate was centrifuged at 15000 ×g for 30 min to remove debris. Total protein concentration was determined using a Micro BCA Protein Assay Kit (Thermo Scientific) (Figures 1G,H). Protein digestion followed the SP3 method [26]: 20 µg of protein from each sample was brought up to 200 µL with Milli‐Q water in 2 mL Safe‐Lock tubes (Ependorf), reduced, and alkylated using 5 mmol L^−1^ dithiothreitol and 15 mmol L^−1^ iodoacetamide, respectively. 10 µL of magnetic bead mixture was added to each sample, followed by 800 µL of HPLC grade acetone. Samples were gently vortexed at room temperature for approximately 10 min, until clumping was observed. Samples were then centrifuged at 5000 × g for 5 min and the supernatant was discarded. Pellets were washed twice with 1 mL of 80% ethanol and gentle pipetting to disrupt the pellet, followed by centrifugation at 5000 ×g for 5 min and removal of supernatant. Final wash supernatant was removed while the tubes were in a magnetic rack to ensure complete solvent removal. 200 µL of 0.5 µg trypsin in 100 mM ammonium bicarbonate and 5 mM CaCl_2_ was then added to each sample, followed by incubation in a Thermocycler (Thermo Scientific) for 16 h at 37°C and 800 RPM. Samples were then centrifuged at 10,000 ×g for 5 min and digested peptides, now contained in supernatant, were transferred to 1.7 mL low binding tubes (Corning). Samples were acidified with 10% TFA and desalted on a StrataX desalt plate (Phenomenex) following manufacturer's guidelines. Clean peptide extracts were brought to dryness and resuspended in 1% formic acid, 3% acetonitrile for liquid chromatography—mass spectrometry (LC‐MS) analysis.

Samples were analyzed on a Waters Acquity M‐Class UPLC coupled to a Thermo Scientific Orbitrap Fusion Lumos Tribrid mass spectrometer. Each sample was measured via three binned injections at a mass range of 430–550 m/z, 550–690 m/z and 690–930 m/z, respectively. By restricting precursor selection to smaller mass windows spanning the full mass range (binning), this method increases sampling depth and maximizes the number of quantifiable peptides and proteins [27]. For each injection, 0.75 µg of peptide digest was separated via one‐dimensional liquid chromatography for 40 min under a non‐linear gradient (Table S1) using a Waters nanoEase M/Z Peptide BEH C18 Column, 130Å, 1.7 µm, 0.075 × 250 mm. The mass spectrometer was operated under positive polarity, data‐independent acquisition mode. (Detailed mass spectrometer settings are shown in Table S2).

Following LC‐MS analysis, Thermo raw files were converted to mzML format using MSConvert with peak picking and demultiplex settings selected. This converts the staggered 4 Da precursor mass window scans to an effective precursor mass window 2 Da. Database searching and peptide quantification was performed with DIA‐NN v1.8.2 [28] using Fragpipe v22.0 [29] against a database combining the full *F. cylindrus* genome [18] and its plastid genome [30], both of which are available on GenBank under accession numbers PRJNA594688 and MK217824.1, respectively. A spectral library was generated using the database and supplemented with database search results. The search output was further processed with the R package iq v1.10.1 to compile protein abundances from the three separate injections and apply MaxLFQ normalization.

We functionally annotated protein coding sequences in the database using eggNOG‐mapper v2 [31, 32]. Of the 18,357 protein coding sequences, 8014 were assigned a functional annotation (Table S3). To quantify *F. cylindrus* protein‐coding genes that have experimental support (mass spectrometry identification), we compared our data with the proteome generated by Kennedy et al. (2019) [24]. We note that our database (18,357 proteins) is different from the one used in [24] (27,137 proteins) because we chose the haploid version and appended plastid‐encoded proteins. To facilitate comparison of the number of identified proteins between studies, we clustered both databases using CD‐HIT [33, 34] at 98% sequence identity (“c” flag), resulting in 24,371 protein clusters.

**FIGURE 2:**
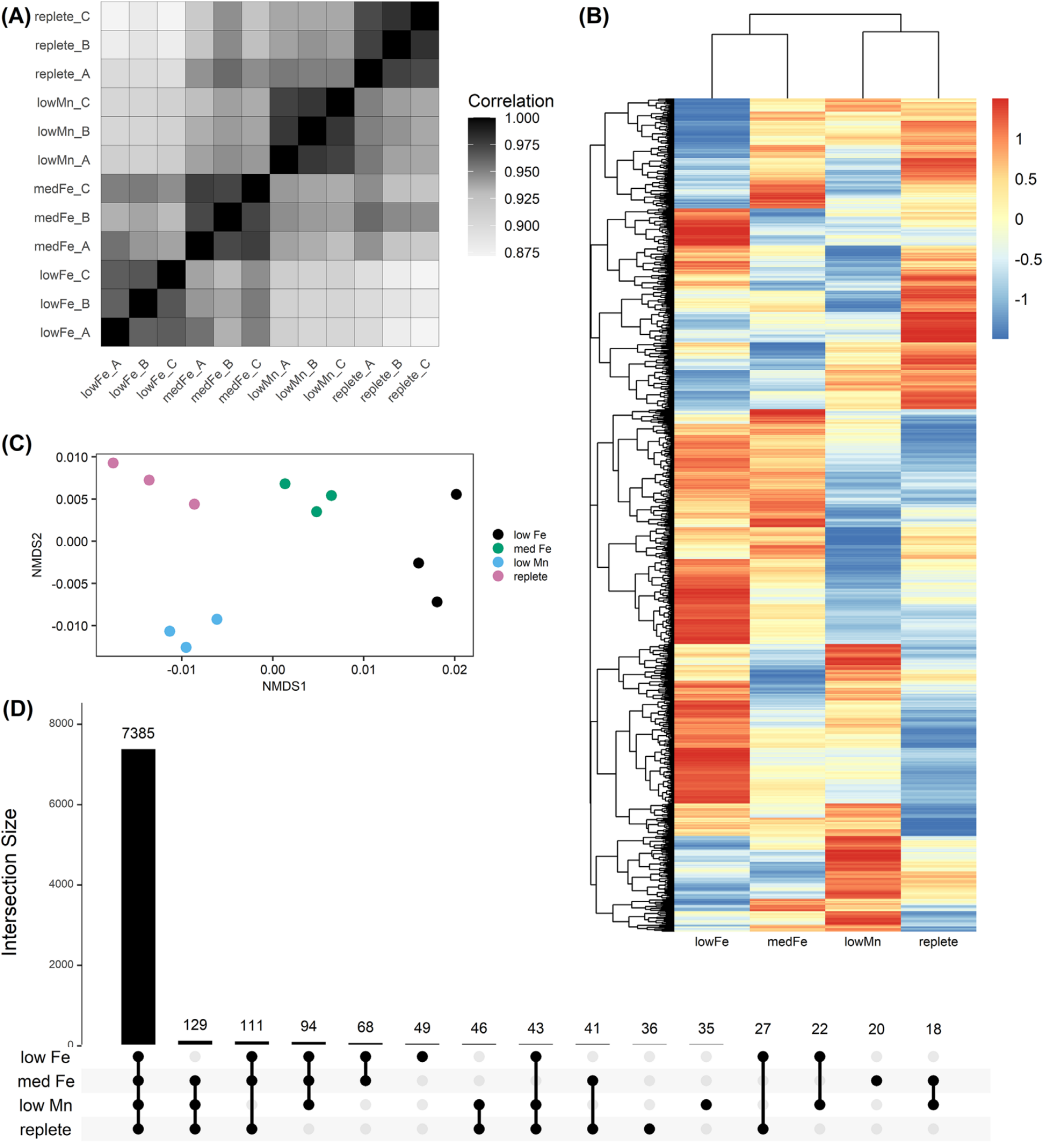
‐ Proteomics data overview. (**A)** Pairwise Pearson correlation matrix of protein abundances across treatments and replicates. (**B)** Heatmap of mean protein abundances in the different treatments. Mean abundances are *z*‐score normalized across treatments, enabling comparisons between treatments but not between proteins. Each row = one unique protein. (**C)** Non‐metric multidimensional scaling (NMDS) ordination of samples based on protein abundance profiles (stress = 0.03). (**D)** UpSet plot summarizing the overlap of proteins measured across treatments.

Following mass spectrometry and data processing, we assessed the reproducibility of our measurements by calculating all possible Pearson correlation coefficients between every sample. This captures both biological variation (e.g., differences within and across treatments) and technical variation (e.g., due to protein extraction and mass spectrometry methods). For the low Fe, medium Fe, low Mn, and replete treatments, the mean Pearson correlation coefficients between biological replicates were 0.95, 0.96, 0.93, 0.96, respectively, indicating a high degree of reproducibility within each treatment (Figure 2A). We also visualized changes in protein abundances across treatments using a heatmap and hierarchical clustering, which highlighted distinct protein expression patterns between treatments (Figure 2B). We then used Bray–Curtis dissimilarity across individual proteomic profiles as inputs for non‐metric multidimensional scaling (NMDS), which showed clustering of replicates within each experimental treatment (Figure 2C). Altogether, these data provide experimental evidence for 59,687 peptides mapping to 8252 proteins, or 45% of the total predicted proteome (18,357 proteins). Most proteins (7385) were present across all treatments, while only small subsets were unique to low iron (49), medium iron (20), low manganese (35) and replete (36) conditions (Figure 2D) (Table S4).

To facilitate comparison with previous proteomic work on *F. cylindrus*, we now switch to reporting the number of protein clusters rather than proteins (because we first needed to cluster the two different databases used for these experiments to identify which were overlapping protein identifications). When compared with Kennedy et al. (2019), we identified 1164 protein clusters in common, they uniquely identified 403 protein clusters, while we uniquely identified 7195 protein clusters. Prior to our dataset, Kennedy et al. (2019) provided experimental support for ∼7% of protein coding genes in *F. cylindrus*, and our dataset adds to this, experimental support for an additional ∼34% of putative protein coding genes in *F. cylindrus*.

**FIGURE 3:**
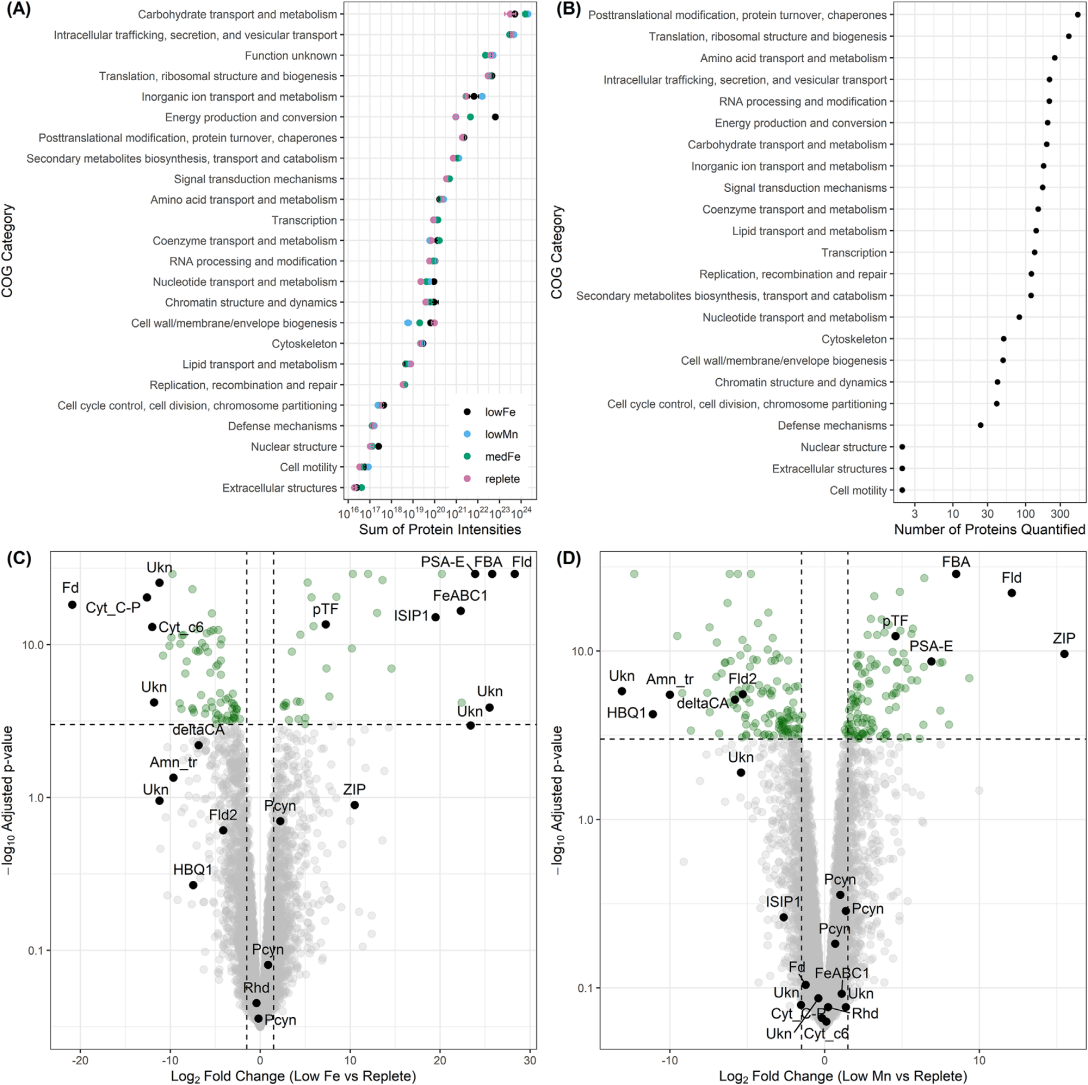
Protein group and differential expression analysis. (**A)** Summed abundances of proteins belonging to various Cluster of Orthologous Group (COG) groups under different treatments. Each point represents the mean of three biological replicates. (**B)** The number of unique proteins belonging to each COG category. Each point represents the total number of proteins in all treatments combined. (**C)** Volcano plot of differential protein expression between low iron and replete treatments. Points above the horizontal dashed line represent statistically significant differences (adjusted *p*‐value <0.05), while the *x*‐axis shows log_2_ fold change (positive = higher abundance under low iron; negative = lower abundance under low iron). (**D)** Volcano plot of differential protein expression between low manganese and replete treatments. Points above the horizontal dashed line represent statistically significant differences (adjusted p‐value <0.05), while the *x*‐axis shows log_2_ fold change (positive = higher abundance under low manganese; negative = lower abundance under low manganese). In C and D, Amn_tr, ammonium transporter; Cyt, cytochrome; deltaCA, delta carbonic anhydrase; FBA, fructose‐bisphosphate aldolase; Fd, ferredoxin; FeABC1, iron ABC transporter; Fld, flavodoxin; ISIP, iron stress induced protein; PCYN, plastocyanin; PSA‐E, peripheral subunit of PSI; pTF, phytotransferrin; Rhd, rhodopsin; Ukn, unknown or proteins of unknown function; ZIP, Zrt‐and Irt‐like protein.

We assigned a Cluster of Orthologous Group (COG) identifier to proteins if they were associated with a single COG category, and identified proteins across various functional groups, particularly those related to photosynthesis, posttranslational modification, protein turnover, chaperones, translation, and amino acid transport and metabolism (Figures 3A,B). We then examined how the protein abundances, with each of these groups, changed across treatments (Figure 3A). Specifically, we summed the median normalized protein abundances within each group. The most abundant protein groups were: “Carbohydrate transport and metabolism”, “Intracellular trafficking”, and “Translation”. Another abundant COG category was “Energy production and conversion”, which was much higher under low iron (Figure 3A).

We performed differential protein expression analysis using the DEP R package (v1.31.0), and applied an adjusted p‐value threshold of 0.05 and log_2_ fold‐change cutoff of 1 to identify significant differences. We used the “normalize_vsn” function within the DEP package to perform variance stabilizing normalization prior to differential expression analysis. In total, 105 proteins were significantly differentially expressed between the low iron and replete treatments, and 229 proteins were significantly differentially expressed between the low manganese and replete treatments (Figure 3C,D) (Table S5). Among the iron responsive proteins, we observed several known signatures of iron stress. Phytotransferrin, other iron stress induced proteins (ISIPs) and iron transporters such as FeABC1 were significantly upregulated under low iron, consistent with their roles in iron acquisition and homeostasis [35, 36, 37, 38]. Plastocyanin, a copper‐containing protein known to substitute iron‐containing cytochrome‐c6 and shown to be upregulated under low iron [39, 40], showed no significant change in abundance under the different iron conditions in our experiment. This aligns with previous findings in Southern Ocean microbial communities [41], and suggests that Southern Ocean phytoplankton may often constitutively express this protein to reduce metabolic iron requirements. Further, two flavodoxin isoforms exhibited contrasting responses to iron: one isoform was strongly iron‐responsive, while the other remained unchanged. This supports the evidence for two flavodoxin clades with divergent functions, only one of which is regulated by iron availability [41, 42, 43]. Flavodoxin is an assumed biomarker of iron stress [44], and our results underscore the importance of distinguishing between isoforms to accurately assess iron stress in marine phytoplankton. Iron‐rich proteins including ferredoxin and cytochrome‐c6 were downregulated under low iron, reflecting a reduction in cellular investment in iron‐containing metalloproteins. We also measured a marked increase in fructose‐bisphosphate aldolase (FBA) under low iron, suggesting a potential role in modulating carbon flux within the chloroplast when energy generation via photosynthesis is restricted (e.g., reduced F_v_/F_m_). This is also supported by transcriptomics‐based measurements showing increased FBA expression during iron limitation [45, 46]. Lastly, PsaE, a component of the iron‐rich photosystem I complex, was upregulated under low iron conditions. PsaE may contribute to stabilizing photosystem I and mitigating reactive oxygen species accumulation when iron is limiting [47]. This response, also observed at the transcript level in *Fragilariopsis kerguelensis* [40] highlights the importance of examining individual protein responses to better understand cellular responses to iron stress, as some responses may appear counterintuitive or may be masked when viewed only at the complex or pathway level.

Among the proteins differentially expressed under varying manganese conditions, HBQ1, a globin‐like protein was down regulated under low manganese (Figure 3D). Although its role in diatoms is not fully resolved [48], in vertebrates this protein is associated with oxygen transport and iron binding, with some evidence suggesting that manganese availability may impact its synthesis and homeostasis [49, 50]. Our findings suggest that manganese may similarly influence these mechanisms in diatoms. Further, delta carbonic anhydrase was downregulated under low manganese, suggesting that manganese may play a role in this enzyme [51]. Interestingly, several proteins upregulated under low iron, including flavodoxin, phytotransferrin, PsaE, and FBA, were also upregulated under low manganese, suggesting *F. cylindrus* employs similar molecular strategies to cope with impaired photosynthetic apparatuses under both iron and manganese limitation. Lastly, we note that many proteins that were differentially expressed under the various iron and manganese conditions were proteins of unknown function. These highlight future opportunities for discovering novel protein functions and molecular responses to trace metal limitation.

This study provides the first proteomic profiles of *Fragilariopsis cylindrus* grown under low iron and manganese conditions like those observed in the Southern Ocean, expanding our understanding of trace metal physiology in Southern Ocean phytoplankton. Beyond its immediate biological insights, this dataset offers a valuable foundation for integration into platforms such as DiatOmicBase and emerging Digital Microbe frameworks [52, 53], enabling cross‐study comparisons and multi‐omics syntheses. Future work examining *F. cylindrus* under simultaneous iron and manganese co‐limitation will be critical for disentangling the shared and unique pathways of trace metal stress responses. Together, these results underscore the utility of proteomics for linking molecular physiology with ecological function in marine ecosystems, and provide a benchmark for future investigations into Southern Ocean biogeochemistry.

## Author Contributions

L.J.J. and E.M.B. designed the culturing experiments and physiological measurements. All authors designed the mass spectrometry analyses. L.J.J. conducted culturing experiments, proteomics sample collection and phenotypic characterizations. E.R. processed the proteomics samples, conducted LC‐MS‐MS measurements, and database searching. L.J.J. and J.S.P.M. analyzed the data. All authors contributed to the writing and editing of the manuscript.

## Conflicts of Interest

The authors declare no conflicts of interest.

## Supporting information




**Supporting File**: pmic70109‐sup‐0001‐tables.xlsx.

## Data Availability

All proteomics data including the raw mass spectrometry files and processed data files have been deposited to the ProteomeXchange Consortium via the PRIDE partner repository [54] with the dataset identifier PXD067269 (username: reviewer_pxd067269@ebi.ac.uk, password: ttvgm0×7PZMi or project accession: PXD067269 and token: Oqug4TdHgI48). All other metadata data are provided as supplemental tables. All code used for data analysis and visualization is available on: https://github.com/LoayJabre/Frag‐Fe‐Mn‐proteome.

## References

[1] C. B. Field , M. J. Behrenfeld , J. T. Randerson , and P. Falkowski , “Primary Production of the Biosphere: Integrating Terrestrial and Oceanic Components,” Science 281 (1998): 237–240. 9657713 10.1126/science.281.5374.237

[2] K. R. Arrigo , “Marine Microorganisms and Global Nutrient Cycles,” Nature 437 (2005): 349–355, 10.1038/nature04159. 16163345

[3] R. W. Eppley and B. J. Peterson , “Particulate Organic Matter Flux and Planktonic New Production in the Deep Ocean,” Nature 282 (1979): 677–680, 10.1038/282677a0.

[4] T. Volk and M. I. Hoffert , in Sundquist ET , Broecker WS (Eds.), Geophys Monogr Ser, American Geophysical Union, Washington, D. C. (1985): 99–110.

[5] H. de Baar , A. Buma , R. Nolting , et al., “On Iron Limitation of the Southern Ocean: Experimental Observations in the Weddell and Scotia Seas,” Marine Ecology Progress Series 65 (1990): 105–122.

[6] J. H. Martin , S. E. Fitzwater , and R. M. Gordon , “Iron Deficiency Limits Phytoplankton Growth in Antarctic Waters,” Global Biogeochemical Cycles 4 (1990): 5–12, 10.1029/GB004i001p00005.

[7] M. Wu , J. S. P. McCain , E. Rowland , et al., “Manganese and Iron Deficiency in Southern Ocean *Phaeocystis antarctica* Populations Revealed Through Taxon‐Specific Protein Indicators,” Nature Communications 10 (2019): 3582, 10.1038/s41467-019-11426-z. PMC6687791 31395884

[8] T. J. Browning , E. P. Achterberg , A. Engel , and E. Mawji , “Manganese Co‐Limitation of Phytoplankton Growth and Major Nutrient Drawdown in the Southern Ocean,” Nature Communications 12 (2021): 884, 10.1038/s41467-021-21122-6. PMC7873070 33563991

[9] J. Balaguer , F. Koch , C. Hassler , and S. Trimborn , “Iron and Manganese Co‐Limit the Growth of Two Phytoplankton Groups Dominant at Two Locations of the Drake Passage,” Communications Biology 5 (2022): 207, 10.1038/s42003-022-03148-8. 35246600 PMC8897415

[10] B. S. Twining and S. B. Baines , “The Trace Metal Composition of Marine Phytoplankton,” Annual Review of Marine Science 5 (2013): 191–215, 10.1146/annurev-marine-121211-172322. 22809181

[11] G. F. de Souza , S. P. Khatiwala , M. P. Hain , S. H. Little , and D. Vance , “On the Origin of the Marine Zinc–silicon Correlation,” Earth and Planetary Science Letters 492 (2018): 22–34, 10.1016/j.epsl.2018.03.050.

[12] P. J. Tréguer , J. N. Sutton , M. Brzezinski , et al., “Reviews and Syntheses: The Biogeochemical Cycle of Silicon in the Modern Ocean,” Biogeosciences 18 (2021): 1269–1289.

[13] R. M. Kellogg , M. A. Moosburner , N. R. Cohen , et al., “Adaptive Responses of Marine Diatoms to Zinc Scarcity and Ecological Implications,” Nature Communications 13 (2022): 1995, 10.1038/s41467-022-29603-y. PMC9010474 35422102

[14] J. Taucher , L. T. Bach , A. E. F. Prowe , T. Boxhammer , K. Kvale , and U. Riebesell , “Enhanced Silica Export in a Future Ocean Triggers Global Diatom Decline,” Nature 605 (2022): 696–700, 10.1038/s41586-022-04687-0. 35614245 PMC9132771

[15] A.‐S. Benoiston , F. M. Ibarbalz , L. Bittner , et al., “The Evolution of Diatoms and Their Biogeochemical Functions,” Philosophical Transactions of the Royal Society B: Biological Sciences 372 (2017): 20160397, 10.1098/rstb.2016.0397. PMC5516106 28717023

[16] P. Tréguer , C. Bowler , B. Moriceau , et al., “Influence of Diatom Diversity on the Ocean Biological Carbon Pump,” Nature Geoscience 11 (2018): 27–37.

[17] S.‐H. Kang and G. A. Fryxell , “ *Fragilariopsis Cylindrus* (Grunow) Krieger: The Most Abundant Diatom in Water Column Assemblages of Antarctic Marginal Ice‐Edge Zones,” Polar Biology 12 (1992): 609–627, 10.1007/BF00236984.

[18] T. Mock , R. P. Otillar , J. Strauss , et al., “Evolutionary Genomics of the Cold‐Adapted Diatom *Fragilariopsis Cylindrus* ,” Nature 541 (2017): 536–540, 10.1038/nature20803. 28092920

[19] T. Mock and N. Hoch , “Long‐Term Temperature Acclimation of Photosynthesis in Steady‐State Cultures of the Polar Diatom *Fragilariopsis Cylindrus* ,” Photosynthesis Research 85 (2005): 307–317, 10.1007/s11120-005-5668-9. 16170633

[20] L. R. Kropuenske , M. M. Mills , G. L. van Dijken , et al., “Strategies and Rates of Photoacclimation in Two Major Southern Ocean Phytoplankton Taxa: Phaeocystis Antarctica (HAPTOPHYTA) and Fragilariopsis Cylindrus (BACILLARIOPHYCEAE) 1,” Journal of Phycology 46 (2010): 1138–1151, 10.1111/j.1529-8817.2010.00922.x.

[21] A. C. Alderkamp , G. Kulk , A. G. J. Buma , et al., “The Effect of Iron Limitation on THE Photophysiology of Phaeocystis Antarctica (PRYMNESIOPHYCEAE) and Fragilariopsis Cylindrus (BACILLARIOPHYCEAE) under Dynamic Irradiance 1,” Journal of Phycology 48 (2012): 45–59, 10.1111/j.1529-8817.2011.01098.x. 27009649

[22] L. Jabre and E. M. Bertrand , “Interactive Effects of Iron and Temperature on the Growth of *Fragilariopsis Cylindrus* ,” Limnology and Oceanography Letters (2020): lol2.10158.

[23] B. R. Lyon , P. A. Lee , J. M. Bennett , G. R. DiTullio , and M. G. Janech , “Proteomic Analysis of a Sea‐Ice Diatom: Salinity Acclimation Provides New Insight Into the Dimethylsulfoniopropionate Production Pathway,” Plant Physiology 157 (2011): 1926–1941, 10.1104/pp.111.185025. 22034629 PMC3327215

[24] F. Kennedy , A. Martin , J. P. Bowman , R. Wilson , and A. McMinn , “Dark Metabolism: A Molecular Insight Into How the Antarctic Sea‐Ice Diatom Fragilariopsis Cylindrus Survives Long‐Term Darkness,” New Phytologist 223 (2019): 675–691, 10.1111/nph.15843. 30985935 PMC6617727

[25] J. S. P. McCain , A. Tagliabue , E. Susko , E. P. Achterberg , A. E. Allen , and E. M. Bertrand , “Cellular Costs Underpin Micronutrient Limitation in Phytoplankton,” Science Advances 7 (2021): abg6501, 10.1126/sciadv.abg6501. PMC8346223 34362734

[26] C. S. Hughes , S. Moggridge , T. Müller , P. H. Sorensen , G. B. Morin , and J. Krijgsveld , “Single‐Pot, Solid‐Phase‐Enhanced Sample Preparation for Proteomics Experiments,” Nature Protocols 14 (2019): 68–85, 10.1038/s41596-018-0082-x. 30464214

[27] C. E. Vincent , G. K. Potts , A. Ulbrich , et al., “Segmentation of Precursor Mass Range Using “Tiling” Approach Increases Peptide Identifications for MS1‐Based Label‐Free Quantification,” Analytical Chemistry 85 (2013): 2825–2832, 10.1021/ac303352n. 23350991 PMC3607285

[28] V. Demichev , C. B. Messner , S. I. Vernardis , K. S. Lilley , and M. Ralser , “DIA‐NN: Neural Networks and Interference Correction Enable Deep Proteome Coverage in High Throughput,” Nature Methods 17 (2020): 41–44, 10.1038/s41592-019-0638-x. 31768060 PMC6949130

[29] F. Yu , G. C. Teo , A. T. Kong , et al., “Analysis of DIA Proteomics Data Using MSFragger‐DIA and FragPipe Computational Platform,” Nature Communications 14 (2023): 4154, 10.1038/s41467-023-39869-5. PMC10338508 37438352

[30] Z. Zheng , H. Chen , and N. Du , “Characterization of the Complete Plastid Genome of Fragilariopsis Cylindrus,” Mitochondrial DNA Part B 4 (2019): 1138–1139, 10.1080/23802359.2019.1586475.

[31] C. P. Cantalapiedra , A. Hernández‐Plaza , I. Letunic , P. Bork , and J. Huerta‐Cepas , “eggNOG‐Mapper v2: Functional Annotation, Orthology Assignments, and Domain Prediction at the Metagenomic Scale,” Molecular Biology and Evolution 38 (2021): 5825–5829, 10.1093/molbev/msab293. 34597405 PMC8662613

[32] J. Huerta‐Cepas , D. Szklarczyk , D. Heller , et al., “eggNOG 5.0: A Hierarchical, Functionally and Phylogenetically Annotated Orthology Resource Based on 5090 Organisms and 2502 Viruses,” Nucleic Acids Research 47 (2019): D309–D314, 10.1093/nar/gky1085. 30418610 PMC6324079

[33] L. Fu , B. Niu , Z. Zhu , S. Wu , and W. Li , “CD‐HIT: Accelerated for Clustering the next‐Generation Sequencing Data,” Bioinformatics 28 (2012): 3150–3152, 10.1093/bioinformatics/bts565. 23060610 PMC3516142

[34] W. Li and A. Godzik , “Cd‐Hit: A Fast Program for Clustering and Comparing Large Sets of Protein or Nucleotide Sequences,” Bioinformatics 22 (2006): 1658–1659, 10.1093/bioinformatics/btl158. 16731699

[35] J. Morrissey , R. Sutak , J. Paz‐Yepes , et al., “A Novel Protein, Ubiquitous in Marine Phytoplankton, Concentrates Iron at the Cell Surface and Facilitates Uptake,” Current Biology 25 (2015): 364–371, 10.1016/j.cub.2014.12.004. 25557662

[36] E. Kazamia , R. Sutak , J. Paz‐Yepes , et al., “Endocytosis‐Mediated Siderophore Uptake as a Strategy for Fe Acquisition in Diatoms,” Science Advances 4 (2018): 15, 10.1126/sciadv.aar4536. PMC5955625 29774236

[37] J. B. McQuaid , A. B. Kustka , M. Oborník , et al., “Carbonate‐Sensitive Phytotransferrin Controls High‐Affinity Iron Uptake in Diatoms,” Nature 555 (2018): 534–537, 10.1038/nature25982. 29539640

[38] J. Behnke and J. LaRoche , “Iron Uptake Proteins in Algae and the Role of Iron Starvation‐Induced Proteins (ISIPs),” European Journal of Phycology 55 (2020): 339–360, 10.1080/09670262.2020.1744039.

[39] G. Peers and N. M. Price , “Copper‐Containing Plastocyanin Used for Electron Transport by an Oceanic Diatom,” Nature 441 (2006): 341–344, 10.1038/nature04630. 16572122

[40] C. M. Moreno , W. Gong , N. R. Cohen , K. DeLong , and A. Marchetti , “Interactive Effects of Iron and Light Limitation on the Molecular Physiology of the Southern Ocean Diatom *Fragilariopsis Kerguelensis* ,” Limnology and Oceanography (2020): lno.11404.

[41] L. J. Jabre , A. E. Allen , J. S. P. McCain , et al., “Molecular Underpinnings and Biogeochemical Consequences of Enhanced Diatom Growth in a Warming Southern Ocean,” Proceedings of the National Academy of Sciences of the United States of America 118 (2021): 2107238118, 10.1073/pnas.2107238118. PMC8325266 34301906

[42] S. Graff van Creveld , S. N. Coesel , S. Blaskowski , and R. D. Groussman , “Divergent Functions of Two Clades of Flavodoxin in Diatoms Mitigate Oxidative Stress and Iron Limitation,” Elife 12 (2023): 84392, 10.7554/eLife.84392. PMC10287166 37278403

[43] L. P. Whitney , J. J. Lins , M. P. Hughes , et al., “Characterization of Putative Iron Responsive Genes as Species‐Specific Indicators of Iron Stress in Thalassiosiroid Diatoms,” Frontiers in Microbiology (2011): 2. 10.3389/fmicb.2011.00234 PMC3223615 22275908

[44] A. Pankowski and A. McMinn , “Development of Immunoassays for the Iron‐Regulated Proteins Ferredoxin and Flavodoxin in Polar Microalgae 1,” Journal of Phycology 45 (2009): 771–783, 10.1111/j.1529-8817.2009.00687.x. 27034052

[45] A. E. Allen , J. LaRoche , U. Maheswari , et al., “Whole‐Cell Response of the Pennate Diatom *Phaeodactylum Tricornutum* to Iron Starvation,” Proceedings of the National Academy of Sciences of the United States of America 105 (2008): 10438–10443, 10.1073/pnas.0711370105. 18653757 PMC2492447

[46] A. E. Allen , A. Moustafa , A. Montsant , A. Eckert , P. G. Kroth , and C. Bowler , “Evolution and Functional Diversification of Fructose Bisphosphate Aldolase Genes in Photosynthetic Marine Diatoms,” Molecular biology and evolution 29 (2012): 367–379, 10.1093/molbev/msr223. 21903677 PMC3245544

[47] R. Jeanjean , A. Latifi , H. C. P. Matthijs , and M. Havaux , “The PsaE Subunit of Photosystem I Prevents Light‐Induced Formation of Reduced Oxygen Species in the Cyanobacterium *Synechocystis* sp. PCC 6803,” Biochimica et Biophysica Acta BBA—Bioenergetics 1777 (2008): 308–316, 10.1016/j.bbabio.2007.11.009. 18164679

[48] C. M. Osuna‐Cruz , G. Bilcke , E. Vancaester , et al., “The Seminavis Robusta Genome Provides Insights Into the Evolutionary Adaptations of Benthic Diatoms,” Nature Communications 11 (2020): 3320, 10.1038/s41467-020-17191-8. PMC7335047 32620776

[49] R. W. Titus and H. W. Cave , “Manganese as a Factor in Hemoglobin Building,” Science 68 (1928): 410–410, 10.1126/science.68.1765.410. 17790941

[50] Y. Liu , J. Hu , R. Tang , et al., “Association Between the Blood Manganese (Mn) and Hemoglobin in Patients Undergoing Maintenance Hemodialysis,” Journal of Trace Elements in Medicine and Biology 71 (2022): 126947, 10.1016/j.jtemb.2022.126947. 35176578

[51] E. L. Jensen , R. Clement , A. Kosta , S. C. Maberly , and B. Gontero , “A New Widespread Subclass of Carbonic Anhydrase in Marine Phytoplankton,” The ISME Journal 13 (2019): 2094–2106, 10.1038/s41396-019-0426-8. 31024153 PMC6776030

[52] I. Veseli , M. A. DeMers , Cooper , et al., “Digital Microbe: A Genome‐Informed Data Integration Framework for Team Science on Emerging Model Organisms,” Scientific Data 11 (2024): 967, 10.1038/s41597-024-03778-z. 39232008 PMC11374999

[53] E. Villar , N. Zweig , P. Vincens , et al., “DiatOmicBase: A Versatile Gene‐Centered Platform for Mining Functional Omics Data in Diatom Research,” Plant Journal 121 (2025): 70061, 10.1111/tpj.70061. PMC11910669 40089834

[54] Y. Perez‐Riverol , J. Bai , C. Bandla , et al., “The PRIDE Database Resources in 2022: A Hub for Mass Spectrometry‐Based Proteomics Evidences,” Nucleic Acids Research 50 (2021): D543–D552, 10.1093/nar/gkab1038. PMC8728295 34723319

